# Berberine Suppresses Influenza A Virus-Triggered Pyroptosis in Macrophages via Intervening in the mtROS-MAVS-NLRP3 Inflammasome Pathway

**DOI:** 10.3390/v17040539

**Published:** 2025-04-07

**Authors:** Mengfan Zhao, Di Deng, Hui Liu, Rui Guo, Jun Wu, Yu Hao, Mingrui Yang

**Affiliations:** School of Life Sciences, Beijing University of Chinese Medicine, Beijing 102488, China; bunianfuhua@163.com (M.Z.); dundee522@163.com (D.D.); 13671081286@163.com (H.L.); ruiguo9@163.com (R.G.); wujunccg1973@163.com (J.W.)

**Keywords:** influenza A virus (IAV), macrophage pyroptosis, berberine (BBR), anti-inflammatory, mitochondrial antiviral signaling protein (MAVS)

## Abstract

Infection with influenza A virus (IAV) may trigger excessive inflammatory responses, leading to severe viral pneumonia and accelerating disease progression. Therefore, controlling these excessive inflammatory responses is crucial for the prevention and treatment of pneumonia caused by IAV. Berberine (BBR), an isoquinoline alkaloid extracted from traditional Chinese medicine, possesses extensive pharmacological activities. However, its immunoregulatory effects and molecular mechanisms in the context of IAV infection require further investigation. This study explored the impact of BBR on macrophage pyroptosis and inflammatory responses induced by IAV infection. Our findings revealed that BBR effectively inhibits the release of IL-1β and TNF-α induced by IAV infection and suppresses gasdermin D (GSDMD)-mediated pyroptosis in a dose-dependent manner. Further research indicates that BBR alleviates macrophage pyroptosis and inflammatory responses in IAV-infected cells by reducing the release of mitochondrial reactive oxygen species (mtROS), inhibiting mitochondrial antiviral signaling protein (MAVS) expression and blocking the activation of the NOD-like receptor family pyrin domain containing 3 (NLRP3) inflammasome. Experiments using siRNA to knockdown MAVS further confirmed the pivotal role of MAVS in BBR’s inhibition of IAV-induced macrophage pyroptosis. This study provides a scientific basis for the application of BBR as an anti-inflammatory drug in the treatment of inflammatory diseases caused by IAV infection and directs future research endeavors.

## 1. Introduction

Influenza viruses, belonging to the Orthomyxoviridae family, are respiratory tract infectious agents categorized into types A, B, and C. Among them, influenza A virus (IAV) is the most prone to mutation, exhibiting high pathogenicity and transmissibility, thus serving as the primary culprit in global influenza pandemics [1,2]. IAV enters into host cells by binding their surface hemagglutinins to sialic acid receptors on respiratory epithelial cells, triggering endocytosis [3]. Once inside, the virus initiates transcription and replication of its genome, producing progeny viruses that subsequently infect additional cells [4]. Concurrently, the inflammatory response mounted by the host immune system contributes to tissue damage and the manifestation of clinical symptoms such as fever, cough, sputum production, chest pain, and dyspnea [5,6]. It has been established that IAV can incite a cytokine storm, accelerating disease progression among infected individuals, leading to viral pneumonia, and in severe cases, hypoxemia and respiratory failure [7]. Notably, similar pathogenic mechanisms have been observed in SARS-CoV-2 induced pneumonia [8,9]. Currently, the treatment strategies for influenza viral pneumonia primarily focus on antiviral therapy and symptomatic management [10]. However, these approaches have shown limited efficacy in reducing severe illness and mortality rates [11]. As such, timely “interruption” of the excessive inflammatory response induced by viral infection holds promise as an effective preventive and therapeutic measure [8].

In the pathogenesis of pneumonia induced by IAV, alveolar macrophages and monocyte-derived macrophages serve as pivotal cells in orchestrating both the innate immune response and excessive inflammatory reactions [12,13]. Upon recognizing viral particles or infected cells, these macrophages initiate localized inflammatory responses by secreting cytokines such as interleukin (IL)-1β, IL-6, and tumor necrosis factor (TNF)-α [14]. Furthermore, macrophages release chemokines such as CCL2 and CCL3 as well as exosomes rich in various chemical signals, guiding the migration of neutrophils and other immune cells toward the site of infection [15]. Apart from the activation and functions of macrophages previously mentioned, the role of macrophage death modalities in the pathogenesis of viral pneumonia has gradually emerged as a focal point of scientific inquiry in recent years [16]. Notably, macrophage pyroptosis, a form of programmed cell death mediated by the NOD-like receptor family pyrin domain containing 3 (NLRP3) inflammasome, plays a pivotal role in the pathogenesis of viral pneumonia [17,18]. Infections such as those caused by IAV can activate the NLRP3 inflammasome, triggering the activation of caspase-1. Activated caspase-1 functions on two fronts: firstly, it cleaves gasdermin D (GSDMD), leading to membrane perforation, cell rupture, and the release of intracellular contents, thereby exacerbating inflammatory responses and tissue damage. Secondly, it processes the precursors of IL-1β and IL-18 into their active forms, which in turn recruit inflammatory cells to the site, amplifying the inflammatory cascade [19,20,21]. Collectively, these mechanisms constitute a complex interaction network of lung macrophages in pneumonia. Research suggests that modulating the signaling pathways and biological functions of lung macrophages may offer novel therapeutic strategies for treating pneumonia [22,23].

Berberine (BBR), an isoquinoline alkaloid extracted from traditional Chinese medicinal herbs, such as *Coptis chinensis* and *Phellodendron amurense*, possesses a broad spectrum of pharmacological activities, encompassing antibacterial, anti-inflammatory, hypoglycemic, hypolipidemic, and anti-apoptotic effects [24,25]. Its anti-inflammatory role in monocytes and macrophages is intimately linked to the suppression of the production and release of proinflammatory cytokines and chemokines including IL-1β, IL-6, and IL-8 [26,27]. Recent research has illuminated the potential therapeutic value of BBR in respiratory diseases, particularly in the treatment of viral pneumonia [28,29,30]. Studies have revealed that BBR, apart from inhibiting viral replication, significantly alleviates inflammatory cell infiltration, inflammatory damage, and edema in the lung tissues of mice with influenza viral pneumonia, thereby reducing the mortality rates [31,32,33,34]. Consequently, BBR has emerged as a potential candidate for interrupting the excessive inflammatory response and disease progression induced by IAV. Our laboratory has conducted preliminary investigations into the mechanisms underlying BBR’s inhibition of influenza viral inflammation. Our findings suggest that BBR’s anti-inflammatory effects in macrophages infected with IAV may be associated with the induction of mitophagy, promoting the clearance of damaged mitochondria, and reducing mitochondrial reactive oxygen species (mtROS) release, thereby inhibiting the excessive activation of the NLRP3 inflammasome in macrophages [35]. Furthermore, BBR has been found to potentially mitigate lung inflammation in mice infected with IAV by modulating the GSDMD-mediated pyroptosis process [36].

Recent studies have demonstrated that mtROS can directly induce the oligomerization of mitochondrial antiviral signaling protein (MAVS), thereby promoting the production of type I interferon (IFN-I), a process that is independent of viral RNA recognition [37]. MAVS, a key protein located on the mitochondrial outer membrane, is capable of recruiting NLRP3 to the mitochondria, thus facilitating the activation of the NLRP3 inflammasome. The activation of the NLRP3 inflammasome is not only regulated by mtROS, but is also closely associated with the exposure of the mitochondrial inner membrane lipid cardiolipin, which translocates to the outer membrane following membrane depolarization and recruits NLRP3 [38,39]. Notably, the activation of the NLRP3 inflammasome can also lead to mitochondrial damage and a further increase in mtROS, creating a positive feedback loop that amplifies the inflammatory response [40]. This intricate interplay indicates that mitochondria are not only upstream regulators of NLRP3 inflammasome activation, but also further promote the amplification of the inflammatory response through the recruitment of MAVS and the production of mtROS. However, the intricate relationship among mtROS, MAVS, NLRP3, and pyroptosis in the context of inflammation induced by IAV as well as the intervening effects of BBR necessitates more systematic and meticulous research. In particular, it is imperative to elucidate the pivotal roles of these molecular mechanisms in modulating the biological behaviors of macrophages via BBR.

In this paper, we detected the intervention effect of BBR on GSDMD-mediated pyroptosis and inflammatory response of mice J774A.1 macrophage induced by the IAV strain and systematically analyzed the relationship between pyroptosis, mtROS, and NLRP3 inflammasome using several molecular specific inhibitors as controls. The data identified that the MAVS serves as a pivotal molecule in the process of pyroptosis induced by IAV, while BBR mitigates excessive inflammation by suppressing the pyroptosis of macrophages infected with IAV via the mtROS-MAVS-NLRP3 inflammasome pathway. This study may shed light on the macrophage-mediated inflammatory biology of IAV infection, reveal the mechanism of BBR as a natural small-molecule drug in interrupting the progression of viral pneumonia, and provide experimental evidence for the clinical application of BBR in the treatment of viral pneumonia.

## 2. Materials and Methods

### 2.1. Cell Lines and Virus

The mouse macrophages (J774A.1 cell line, Procell Life Science & Technology Company, Wuhan, China) were cultured into high-glucose DMEM (No. XB01-02, HyClone, Logan, UT, USA) containing 10% fetal bovine serum (FBS, No. 16000044, Gibco, Grand Island, NY, USA) and 1% antibiotic penicillin-streptomycin (No. SV30010, HyClone, Logan, UT, USA) at 37 °C under 5% CO_2_. The mouse-adapted IAV PR8 strain (strain A/Puerto Rico/8/1934 (H1N1)) was amplified from the allantois of 9-day-old chicken embryos at 35 °C for 48 h, then at 4 °C for 12 h, and stored at −80 °C in our laboratory. The titer of virus was 10^−5.4^/0.1 mL in the J774A.1 cells using 50% tissue culture infectious dose (TCID_50_) as the standard.

### 2.2. Virus Infection and Cell Treatment

J774A.1 cells were seeded in six-well plates (1 × 10^6^ cells/well) and infected or treated when the density reached more than 60%. The cells were infected with the 100 TCID_50_ PR8 strain in serum-free DMEM for 6 h, 12 h, or 24 h. BBR chloride (No. 110713-201413, The National Institute for Food and Drug Control, Beijing, China) was used at concentrations of 4.2 μM, 8.4 μM, or 16.8 μM to treat cells for 24 h, consistent with previously published studies [35]. These concentrations all ensured a cell survival rate of over 90%. MCC950 (No. CP-456773, Selleckchem, Houston, TX, USA), a potent and selective NLRP3 inhibitor, was utilized to pretreat cells at a concentration of 50 µM, 3 h prior to viral infection. Monosodium urate (MSU, No. U2875, Sigma, St. Louis, MO, USA), a NLRP3 inflammasome activator, was employed to stimulate cells at a concentration of 150 µg/mL, followed by subsequent treatment with BBR. N-acetyl-L-cysteine (NAC, No. S0077, Beyotime, Shanghai, China), a direct antioxidant with a strong ROS-scavenging effect, was used to pretreat cells at a concentration of 500 µM for 1 h prior to infection with PR8.

### 2.3. Enzyme-Linked Immunosorbent Assay (ELISA)

Detection was performed using ELISA kits, employing a sandwich method for lactate dehydrogenase (LDH) (No. KGT02424, KeyGEN BioTECH, Nanjing, China) and interleukin (IL)-1β (No. XY-R0012c, Biolegend, San Diego, CA, USA). The supernatant was diluted and added to the pre-coated microtiter plate, followed by a 30-minute incubation at 37 °C. After washing, enzyme-labeled reagents were added and incubated for another 30 min at 37 °C. Subsequently, a chromogenic solution was added and allowed to react for 10 min at 37 °C. Finally, a stop solution was added, and the optical density (OD) values were measured using a microplate reader (SpectraMax i3x, No. GF3637001, Molecular Devices, San Jose, CA, USA) at a wavelength of 450 nm.

### 2.4. Western Blot Assay

Cells were collected and lysed with RIPA lysate (No. R0010, Solarbio, Beijing, China) with protease inhibitor, centrifuged at 12,000 rpm for 10 min at 4 °C, and the resulting supernatant was collected. The Bicinchoninic Acid (BCA) Protein Assay Kit (No. P0112, Beyotime, Shanghai, China) was applied to quantify the protein. The proteins were separated by 10–15% SDS-PAGE electrophoresis after denaturation and transferred to polyvinylidene fluoride (PVDF) membranes (No. ISEQ00010, Millipore, Bedford, MA, USA), and the PVDF was blocked in TBST (No.T1085, Solarbio, Beijing, China) solution with 5% fat-free milk (No. 9999S, CST, Danvers, MA, USA) for 1 h and incubated with the following primary antibodies at 4 °C overnight, respectively: anti-NLRP3 antibody (1:1000, No. ab16097, Abcam, Cambridge, UK), anti-GSDMD-N antibody (1:1000, No. sc-393656, Santa Cruz, Dallas, TX, USA), anti-MAVS antibody (1:1000, No. ab189109, Abcam, Cambridge, UK), and anti-β-actin antibody (1:5000, No. 66009-1-Ig, Proteintech, Wuhan, China). The membrane was washed with 10 mL TBST buffer three times, followed by incubating the secondary antibodies (1:5000, goat anti-mouse, No. SA00001-1 and rabbit anti- mouse, No. SA00001-2, Proteintech, Wuhan, China), which were conjugated with horseradish peroxidase (HRP) under room temperature for 1 h. Then, the blots were detected with an ECL reagent (No. A38554, Thermo Fisher, Waltham, MA, USA) after being washed three times with TBST and visualized using the ChemiDoc MP Imaging System (No.12003154, Bio-Rad, Hercules, CA, USA). The densitometry of the immunoblot was calculated using ImageJ software (v1.53j), and the ratio of the target to internal reference β-actin was presented.

### 2.5. Flow Cytometry

The FAM-FLICA Caspase-1 Kit (No. ICT097, Bio-Rad, Hercules, CA, USA) was employed to measure the rate of caspase-1-mediated cell pyroptosis. The fluorescently labeled caspase inhibitor (FLICA) within the kit specifically binds to the active site of the activated caspase-1 enzyme, thereby enabling the detection of caspase-1 activity via FLICA. The simplified steps are as follows: cells collected from a 6-well plate were washed twice with PBS, then suspended in FAM-YVAD-FMK (diluted 1:300) and incubated at 37 °C in the dark for 1 h. Following this, the cells were washed twice with FLICA buffer and resuspended in propidium iodide (2 µg/mL) to detect dead cells. Finally, the samples were analyzed using a flow cytometer (CytoFLEX, No. D00796, Beckman Coulter, Brea, CA, USA) and FlowJo software (v10.6.1) for data interpretation.

To assess the generation of reactive oxygen species (ROS), cells were stained with the fluorescent probe 2-7′-dichlorodihydrofluorescein diacetate (DCFH-DA, No. MX4802, Maokang, Shanghai, China) at a concentration of 2 µM and incubated at 37 °C for 1 h in a light-protected environment. Following incubation, the cells were washed twice with phosphate-buffered saline (PBS) and analyzed using flow cytometry. Data analysis was performed utilizing FlowJo software (v10.6.1).

### 2.6. Quantitative Real-Time Polymerase Chain Reaction (RT-qPCR)

The cells in each group were harvested to make the cell suspension and treated by TRIzol (No. 15596-026, Ambion, Austin, TX, USA) to extract the total RNA. The purity and integrity of the extracted RNA were carefully analyzed to assess its quality and quantity. After determining the RNA concentration, the total RNA (1 μg) was subjected to reverse transcription using a Transcriptor First Strand cDNA Synthesis Kit (No. FSK-101, TOYOBO, Osaka, Japan) following the manufacturer’s instructions. Quantitative real-time PCR was performed using the CFX96 system (No. 1855095, Bio-Rad, Hercules, CA, USA) and SYBR Green Mix Kit (No. RT210, TIANGEN, Beijing, China). Amplification was performed at 50 °C for 2 min and 95 °C for 10 min, followed by 40 cycles of 95 °C for 15 s, 60 °C for 15 s, and 72 °C for 30 s. The relative mRNA expression levels were normalized to GAPDH using the 2^−ΔΔCt^ method, and the results were analyzed with QuantStudio Real-Time PCR software (v1.3). Primers used in the study are listed in Supplementary Table S1 and synthesized by Sangon Biotech (Shanghai, China).

### 2.7. Immunofluorescence Under Confocal Laser Scanning Microscopy

Cells were initially seeded in glass-bottomed confocal chambers, with an approximate density of 1×10^6^ cells per chamber. These were then cultured overnight to facilitate adhesion, followed by infection with the PR8 virus and subsequent treatment with BBR for a duration of 24 h. After this period, the cells were fixed using 4% paraformaldehyde (PFA) and permeabilized with 0.3% Triton X-100. Finally, they were thoroughly washed three times with cold PBS buffer. Prior to the application of the anti-GSDMD-N antibody (1:200, No. sc-393656, Santa Cruz, Dallas, TX, USA), anti-NLRP3 antibody (1:200, No. ab16097, Abcam, Cambridge, UK), anti-MAVS (1:200, No. ab189109, Abcam, Cambridge, UK), and anti-ASC antibody (1:200, No. 04-147, Merck Millipore, Billerica, MA, USA), the cells underwent a blocking step using PBS supplemented with 5% goat serum (No.ZLI-9022, BIODEE, Beijing, China) for 1 h at room temperature. Following an overnight incubation with the primary antibodies at 4 °C, the cells were rinsed three times with PBS. Subsequently, they were co-incubated with goat anti-mouse conjugated with Alexa Fluor 647 (for GSDMD-N, red, No. ab150115, Abcam, Cambridge, UK), donkey anti-rabbit conjugated with FITC (for MAVS, green, No. AS042, ABclonal, Woburn, MA, USA), goat anti-mouse conjugated with Dylight 405 (for NLRP3 and ASC, blue, No. ab175660, Abcam, Cambridge, UK) secondary antibodies, respectively, in a dark environment at room temperature for 1 h. After washing, DAPI solution (No. C1005, Beyotime, Shanghai, China) was administered for nuclear staining at room temperature for 10 min, Mito Tracker Red CMXROS (100 nM, No. M7512, Thermo Fisher, Waltham, MA, USA) was for mitochondria observation, and DCFH-DA was for ROS (No. MX4802, Maokang, Shanghai, China) marking at 37 °C for 30 min, followed by washing three times using PBS buffer. The expression and localization of targets were detected by immunofluorescence analysis under an FV3000 confocal laser scanning microscope (Olympus, Tokyo, Japan,) with oil immersion and 100 × objective in 1 mL PBS buffer.

### 2.8. Small-Interfering RNA (siRNA) Transfection for Gene Silencing

The MAVS-siRNA and control-siRNA used in the study are listed in Supplementary Table S2 and the powders (No. 68042, Santa Cruz, Dallas, TX, USA) were dissolved in RNase-free water according to the recommended dosages provided in the instructions. Subsequently, the cell culture medium was replaced with serum-free Opti-MEM medium (No. 31985070, HyClone, Logan, UT, USA). In compliance with the Lipofectamine™ 2000 (No. 11668019, Thermo Fisher, Waltham, MA, USA) protocol, the siRNA solutions were mixed with the transfection reagent to formulate the transfection complexes, which were then gently pipetted into the wells containing cells to ensure uniform distribution. The plate was incubated in a cell culture incubator for 5 h, followed by a medium exchange with serum-containing medium for a further 24-h culture.

### 2.9. Quantification and Statistical Analysis

Statistical analysis was performed with GraphPad Prism version 8.3.0 (GraphPad Software, Inc., San Diego, CA, USA). The experimental results are shown as the mean ± SD and the ANOVA test was used for comparison between groups. Differences were considered statistically significant if the *p*-value < 0.05. Significance levels were: * *p* < 0.05 and ** *p* < 0.01 vs. the mock group; # *p* < 0.05; and ## *p* < 0.01 vs. the PR8 group. Each set of experiments was independently repeated three times.

## 3. Results

### 3.1. IAV Induces NLRP3-Caspase-1 Mediated Pyroptosis in Macrophages in a Time Dependent Manner

**Figure 1:**
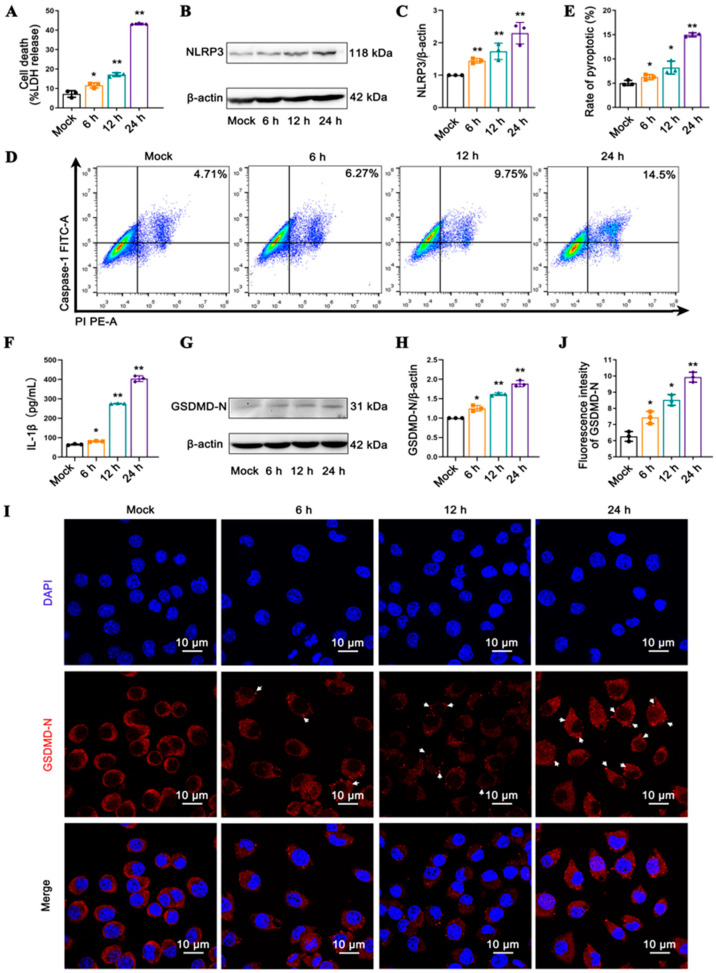
IAV induces NLRP3-caspase-1 mediated pyroptosis in macrophages in a time-dependent manner. J774A.1 cells uninfected and infected with the PR8 virus for 6 h, 12 h or 24 h. (**A**) The LDH levels in the culture supernatants from dead cells were measured using ELISA. (**B**,**C**) The expression levels of NLRP3 were detected by Western blot using β-actin as an internal control. (**D**,**E**) Flow cytometric analysis of the rates of pyroptosis mediated by caspase-1 using the FAM-FLICA Caspase-1 Kit. (**F**) The IL-1β levels in the culture supernatants were measured using ELISA. (**G**,**H**) The expression levels of GSDMD-N were detected by Western blot using β-actin as an internal control. (**I**) Representative images of immunofluorescence staining for GSDMD-N were observed by confocal laser scanning microscope. The nuclei are shown in blue using DAPI staining; while GSDMD-N is shown in red and detected by antibody. The white arrows indicate the fluorescent hub formed by GSDMD-N aggregation. (**J**) Bar diagram of the quantitative summary for GSDMD-N immunofluorescence. Around sixty cells in each group from three graphs were counted. * *p* < 0.05, ** *p* < 0.01 vs. the mock group.

To elucidate the association between the duration of IAV infection and the magnitude of pyroptosis induced by the virus, J774A.1 macrophages were infected with the IAV PR8 strain for 6, 12, and 24 h, respectively. As the duration of infection progressed, the level of LDH release gradually escalated (Figure 1A), indicative of a heightened degree of cellular damage. Concurrently, a gradual increase in the expression levels of NLRP3 (Figure 1B,C), the percentage of cells doubly labeled with caspase-1 and PI (Figure 1D,E) as well as the amount of IL-1β (Figure 1F) released were observed. These suggest that the production of NLRP3 inflammasome and its downstream effectors, caspase-1 and IL-1β, is positively correlated with the duration of viral infection. The WB and immunofluorescence analysis revealed a marked increase in the level of GSDMD-N (Figure 1G–J), particularly highlighting the conspicuous hubs formed by the polymerization of GSDMD-N localized to the cell membrane (Figure 1I,J), reflecting the level of GSDMD-mediated pyroptosis. Furthermore, we observed a positive correlation between the mRNA transcription levels of NLRP3, caspase-1, and GSDMD and the duration of viral infection (Figure S1A–C). These findings suggest that IAV elicits the activation of the NLRP3 inflammasome in macrophages, thereby promoting the cleavage of GSDMD, mediating the onset of pyroptosis and ultimately leading to an exaggerated inflammatory response in a time-dependent manner. In subsequent experiments, we chose the 24-h time point when PR8 virus infection induced the strongest pyroptosis for research.

### 3.2. BBR Inhibited GSDMD-Mediated Pyroptosis and Cytokine Release in Macrophages After IAV Infection

**Figure 2:**
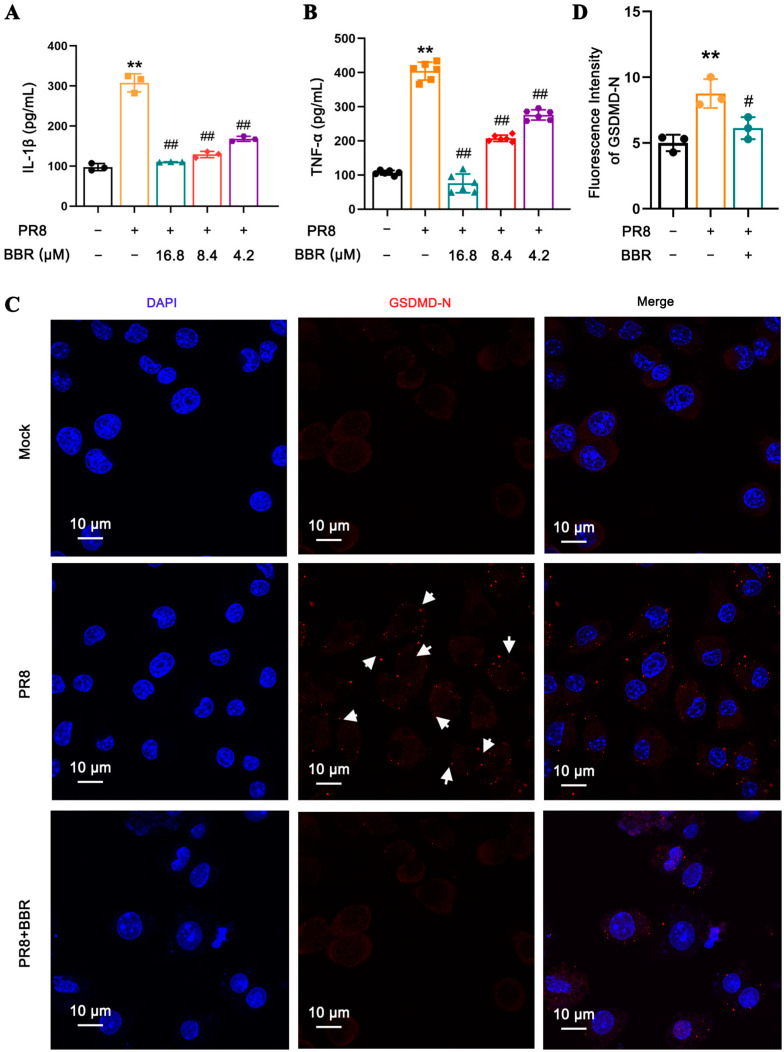
BBR inhibited GSDMD-mediated pyroptosis and cytokine release in macrophages infected with IAV. (**A**) The IL-1β and (**B**) TNF-α release levels in the culture supernatants from J774A.1 cells uninfected, infected with PR8 for 24 h, and infected with PR8 combined with BBR treatment at concentrations of 4.2, 8.4, and 16.8 μM were measured using ELISA. (**C**) Immunofluorescence staining for GSDMD-N was observed by confocal laser scanning microscope. J774A.1 cells were treated with the PR8 virus while BBR was at a concentration of 16.8 μM for 24 h. The nuclei are shown in blue using DAPI staining; GSDMD-N is shown in red and detected by antibody. The white arrows indicate the fluorescent hub formed by GSDMD-N aggregation. (**D**) Bar diagram of the quantitative summary for GSDMD-N immunofluorescence. Around sixty cells in each group from three graphs were counted. ** *p* < 0.01 vs. the mock group. # *p* < 0.05, ## *p* < 0.01 vs. the PR8 group.

In order to further study the effect and mechanism of BBR on IAV-induced pyroptosis, low (4.2 μM), medium (8.4 μM), and high (16.8 μM) doses of BBR, which ensured a cell survival rate of more than 90%, were used in the experiment. The results showed that BBR could significantly inhibit the levels of IL-1 and TNFα released from the macrophages infected by PR8 in a dose-dependent manner (Figure 2A,B). Therefore, BBR at 16.8 μM with the best anti-inflammatory effect was selected for the subsequent experiments. The inhibitory effect of BBR on the expression of PR8-activated GSDMD-N was determined by the immunofluorescence assay (Figure 2C,D). Notably, we also observed that the localization of GSDMD-N was very close to that of mitochondria (Figure S2A,B), suggesting a potential association between GSDMD activity and mitochondrial function.

### 3.3. BBR Attenuates NLRP3 Inflammasome Activation to Reduce Macrophages Pyroptosis Triggered by IAV

**Figure 3:**
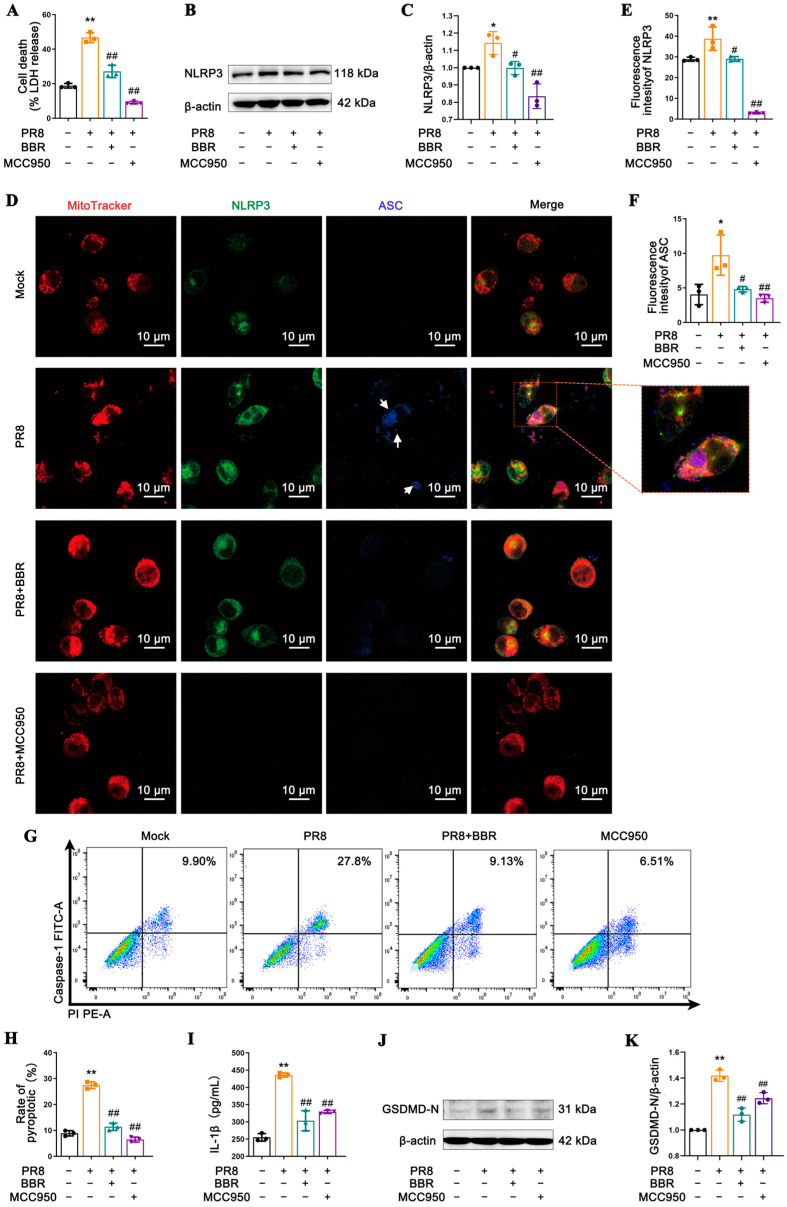
BBR attenuates NLRP3 inflammasome activation to reduce macrophage pyroptosis triggered by IAV. J774A.1 cells were infected with the PR8 virus for 24 h in the presence of BBR (16.8 µM) treatment. As a positive control, cells were pretreated with the NLRP3 inhibitor MCC950 (50 μM) for 3 h and then infected with the PR8 virus for an additional 24 h. (**A**) The LDH levels in the culture supernatants from the dead cells were measured using ELISA. (**B**,**C**) The expression levels of NLRP3 were detected by Western blot using β-actin as an internal control. (**D**) Immunofluorescence staining for the NLRP3 inflammasome was observed by confocal laser scanning microscope. NLRP3 and ASC are shown in green and blue, detected by antibody, respectively, while the mitochondria are shown in red using Mito Tracker staining. The white arrows represent regions with ASC expression. (**E**) Bar diagram of the quantitative summary for the NLRP3 immunofluorescence. (**F**) Bar diagram of the quantitative summary for ASC immunofluorescence. Around sixty cells in each group from three graphs were counted. (**G**,**H**) Flow cytometric analysis of the rates of pyroptosis mediated by caspase-1 using the FAM-FLICA Caspase-1 Kit. (**I**) The IL-1β levels in the culture supernatants were measured using ELISA. (**J**,**K**) The expression levels of GSDMD-N were detected by Western blot using β-actin as an internal control. * *p* < 0.05, ** *p* < 0.01 vs. the mock group. # *p* < 0.05, ## *p* < 0.01 vs. the PR8 group.

To investigate the effects of BBR on NLRP3 inflammasome activation and pyroptosis induced by IAV in a macrophage model, we performed experiments using MCC950 (50 μM), a specific NLRP3 inhibitor, as the control. We observed that both BBR and MCC950 effectively suppressed the PR8-induced LDH release in the J774A.1 macrophages, indicating their inhibitory effect on virus-induced cell damage (Figure 3A). Consistent with the biological effects of MCC950, WB and immunofluorescence analyses confirmed that BBR also significantly inhibited the expression of NLRP3 (Figure 3B–E) and ASC (Figure 3D,F), reflecting its inhibitory effect on NLRP3 inflammasome activation. Furthermore, BBR and MCC950 reduced caspase-1-mediated pyroptosis (Figure 3G,H) as well as the release of cytokines IL-1β (Figure 3I) and GSDMD-N (Figure 3J,K) as downstream of caspase-1. These results indicate that BBR exhibits similar effects to MCC950 in macrophages, affecting NLRP3 inflammasome activation induced by IAV and downstream caspase-1 cleavage, intervening in GSDMD-mediated pyroptosis and the release of inflammatory cytokines such as IL-1β. Additionally, the high colocalization of NLRP3, ASC, and Mito Tracker suggests that BBR’s anti-pyroptosis effect may be related to the regulatory role of mitochondria in NLRP3 inflammasome activation (Figure 3D).

### 3.4. The Inhibitory Effect of BBR on Pyroptosis Mediated by the NLRP3 Inflammasome in Macrophages Is Related to MAVS

**Figure 4:**
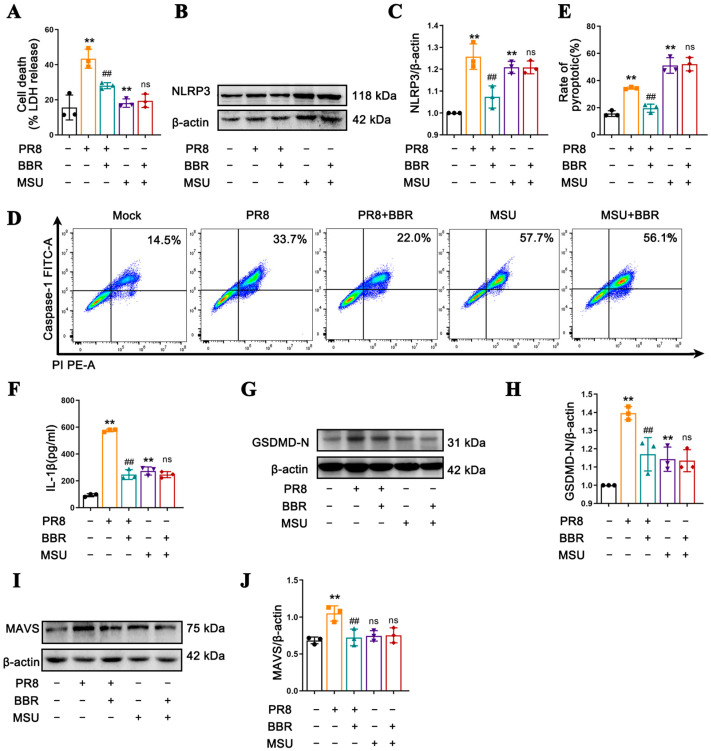
The inhibitory effect of BBR on pyroptosis mediated by the NLRP3 inflammasome in macrophages is related to MAVS. J774A.1 cells were infected with PR8 virus for 24 h in the presence of BBR (16.8 µM) treatment while cells were stimulated with the NLRP3 inflammasome activator MSU (150 µg/mL) for 24 h as a control. (**A**) The LDH levels in the culture supernatants from dead cells were measured using ELISA. (**B**,**C**) The expression levels of NLRP3 were detected by Western blot using β-actin as an internal control. (**D**,**E**) Flow cytometric analysis of the rates of pyroptosis mediated by caspase-1 using the FAM-FLICA Caspase-1 Kit. (**F**) The IL-1β levels in the culture supernatants were measured using ELISA. (**G**,**H**) The expression levels of GSDMD-N were detected by Western blot using β-actin as an internal control. (**I**,**J**) The expression levels of MAVS were detected by Western blot using β-actin as an internal control. ** *p* < 0.01 vs. the mock group. ## *p* < 0.01 vs. the PR8 group. ns represents no statistical significance vs. the MSU group.

Based on the established link between BBR’s inhibition of NLRP3 inflammatory activation and its interference with mitochondrial localization, we aimed to determine whether BBR exerted its suppressive effect on NLRP3 inflammasome activation through MAVS, which functions as a crucial step in recruiting NLRP3 to mitochondria for its activation. We opted to utilize MSU, a crystalline activator that can directly activate the NLRP3 inflammasome without involving MAVS, to treat cells as a control group. The results demonstrated that both the PR8 virus and MSU treatment alone significantly increased the release of LDH (Figure 4A), the expression of NLRP3 (Figure 4B,C), rate of caspase-1-mediated pyroptosis (Figure 4D,E), release of IL-1β (Figure 4F), and level of GSDMD-N (Figure 4G,H) in the cells. However, the treatment of BBR significantly reversed the enhancement of these indicators induced by PR8 while having almost no effect on the effects of MSU (Figure 4A–H). Concurrently, it was observed that BBR also suppressed the elevation of the NLRP3, caspase-1, and GSDMD mRNA levels induced by PR8 while having no effect on MSU’s transcriptional enhancement (Figure S3A–C). Subsequently, we examined the expression levels of MAVS in different groups and discovered that PR8 significantly promoted the increase in the MAVS levels whereas MSU did not. Notably, BBR treatment significantly suppressed the MAVS levels in both the PR8- and MSU-treated cells (Figure 4I,J). These findings suggest that BBR may inhibit the activation of the NLRP3 inflammasome on mitochondria by downregulating the expression of MAVS protein, thereby interfering with PR8-induced macrophage pyroptosis.

### 3.5. BBR Suppresses Mitochondrial ROS Release in Macrophages Infected with IAV

**Figure 5:**
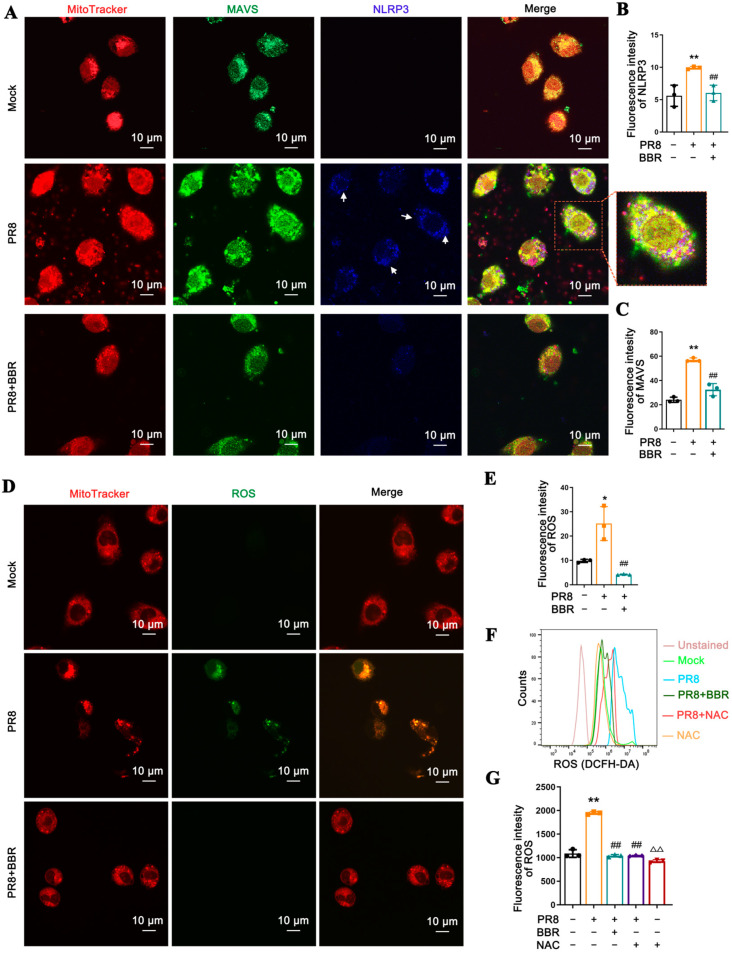
BBR suppresses mitochondrial ROS release in macrophages infected with IAV. (**A**) Immunofluorescence staining for MAVS was observed by confocal laser scanning microscope. MAVS and NLRP3 are shown in green and blue, detected by antibody, respectively, while the mitochondria are shown in red using Mito Tracker staining. The white arrows represent regions with higher NLRP3 expression. (**B**) Bar diagram of the quantitative summary for NLRP3 immunofluorescence. (**C**) Bar diagram of the quantitative summary for MAVS immunofluorescence. (**D**) Immunofluorescence staining for mtROS was observed by confocal laser scanning microscope. The ROS are shown in green marked by DCFH-DA, while the mitochondria are shown in red using Mito Tracker staining. The white arrows represent regions with higher NLRP3expression. (**E**) Bar diagram of the quantitative summary for ROS immunofluorescence. (**F**,**G**) Detection of the mtROS levels by flow cytometry with DCFH-DA. * *p* < 0.05, ** *p* < 0.01 vs. the mock group. ## *p* < 0.01 vs. the PR8 group. ^△△^ *p* < 0.01 vs. the PR8 + NAC group.

Immunofluorescence experiments have validated that BBR exerts inhibitory effects on the level of MAVS and the recruitment of NLRP3 in mitochondria induced by PR8 (Figure 5A–C). Given that mitochondrial ROS (mtROS) serves as a crucial signal in regulating the activation of NLRP3 inflammasomes, we examined the impact of PR8 stimulation and BBR treatment on the mtROS levels in macrophages. The immunofluorescence experiments revealed a significant inhibitory effect of BBR on the elevation of mitochondrial ROS in the PR8-induced cells (Figure 5D,E). Flow cytometry analysis also yielded consistent results, suggesting that BBR’s inhibitory effect on mtROS is analogous to that of the mitochondrial antioxidant Mito-TEMPO (Figure 5F,G).

### 3.6. BBR Inhibits Expression of MAVS and NLRP3 Inflammasome Activation in Macrophages with IAV by Reducing mtROS

**Figure 6:**
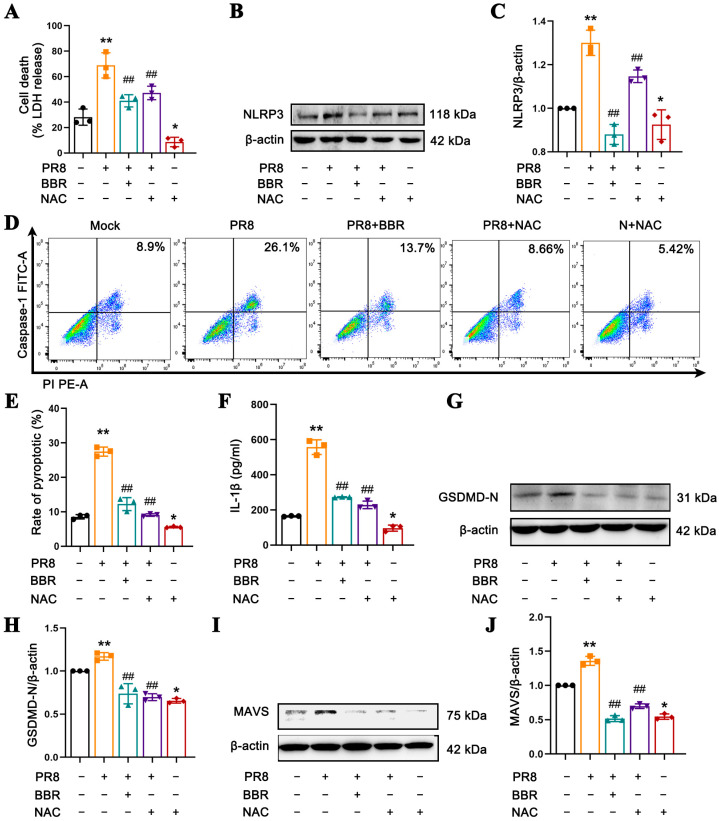
BBR inhibits the expression of MAVS and NLRP3 inflammasome activation in macrophages with IAV by reducing mtROS. J774A.1 cells were infected with the PR8 virus for 24 h in the presence of BBR (16.8 µM) treatment. As a positive control, cells were pretreated with ROS scavenger NAC (500 µM)) for 1 h and then infected with the PR8 virus for an additional 24 h. (**A**) The LDH levels in the culture supernatants from dead cells were measured using ELISA. (**B**,**C**) The expression levels of NLRP3 were detected by Western blot using β-actin as an internal control. (**D**,**E**) Flow cytometric analysis of the rates of pyroptosis mediated by caspase-1 using the FAM-FLICA Caspase-1 Kit. (**F**) The IL-1β levels in the culture supernatants were measured using ELISA. (**G**,**H**) The expression levels of GSDMD-N were detected by Western blot using β-actin as an internal control. (**I**,**J**) The expression levels of MAVS were detected by Western blot using β-actin as an internal control. * *p* < 0.05, ** *p* < 0.01 vs. the mock group. ## *p* < 0.01 vs. the PR8 group.

To elucidate the effect of BBR on the release of mtROS and the correlation between mtROS, MAVS, and NLRP3 inflammasome, we employed the specific ROS scavenger NAC to limit ROS release as the control. When compared with the PR8-only stimulated group, the BBR and NAC treatments significantly reduced the release of LDH release (Figure 6A), the expression of NLRP3 (Figure 6B,C), the rate of caspase-1-mediated pyroptosis (Figure 6D,E), the secretion of IL-1β (Figure 6F), the level of GSDMD-N (Figure 6G,H), and the expression of MAVS (Figure 6I,J). These results suggest that BBR exerts a similar mtROS scavenging effect as NAC, reducing MAVS expression on the mitochondrial membrane, blocking the translocation of NLRP3 from the cytoplasm to mitochondria, and the binding of NLRP3 to MAVS, thereby inhibiting the activation of the NLRP3 inflammasome. This ultimately reduces pyroptosis in the macrophages and inhibits excessive inflammatory responses induced by IAV.

### 3.7. MAVS Is a Crucial Molecule in the Progress of BBR Inhibiting IAV-Triggered Pyroptosis in Macrophages

**Figure 7:**
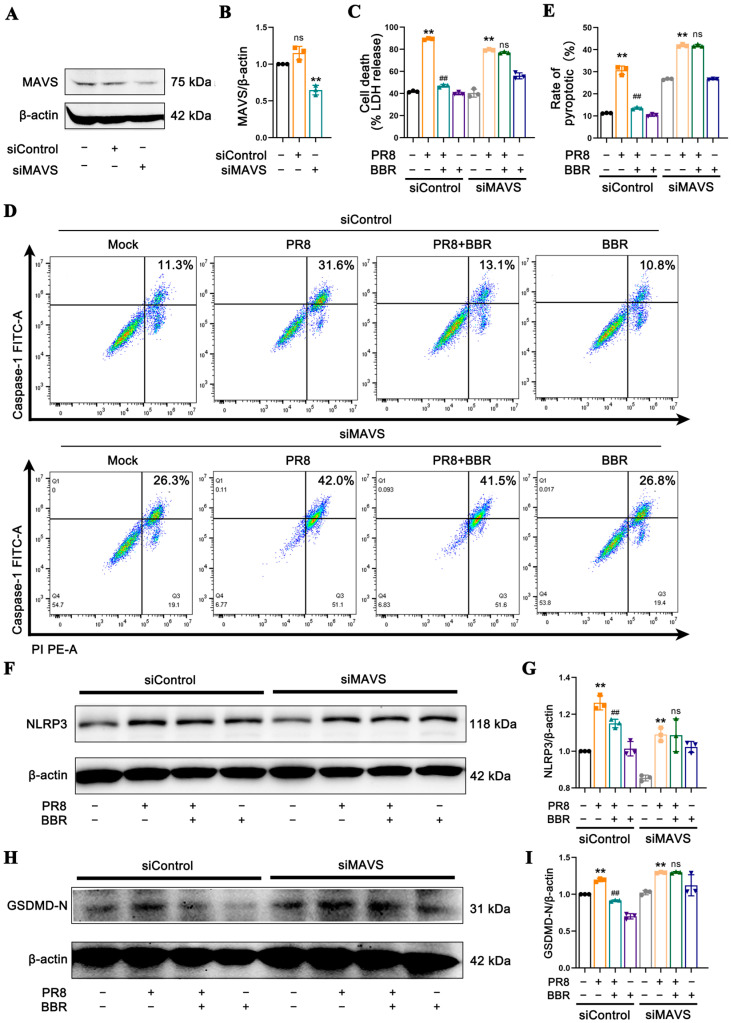
Validation of the role of MAVS in BBR inhibiting pyroptosis induced by IAV using knockdown. After being transfected with siRNAs targeting MAVS or the control siRNA, cells were infected with PR8 in the presence or absence of BBR (16.8 µM) treatment for 24 h. (**A**,**B**) The expression levels of MAVS were detected by Western blot using β-actin as an internal control. (**C**) The LDH levels in the culture supernatants from dead cells were measured using ELISA. (**D**,**E**) Flow cytometric analysis of the rates of pyroptosis mediated by caspase-1 using the FAM-FLICA Caspase-1 Kit. (**F**,**G**) The expression levels of NLRP3 were detected by Western blot using β-actin as an internal control. (**H**,**I**) The expression levels of GSDMD-N were detected by Western blot using β-actin as an internal control. ** *p* < 0.01 vs. the mock group. ## *p* < 0.01 vs. the PR8 group. ns represents no statistical significance vs. the MSU group.

To further elucidate the role of MAVS in BBR’s suppression of macrophage pyroptosis, we employed siRNA to silence MAVS and assessed whether BBR could still exert its effects. After determining the knockdown effect of siRNA on MAVS (Figure 7A,B), it was found that the PR8 virus remained capable of activating NLRP3 inflammasome-mediated apoptosis, resulting in elevated LDH release levels (Figure 7C), pyroptosis mediated by caspase-1 (Figure 7D,E), and increased levels of NLRP3 (Figure 7F,G) and GSDMD-N (Figure 7H,I) compared with the control group. However, BBR no longer exerted an inhibitory effect, and there were no statistically significant differences in the release and expression of pyroptosis-related molecules between the BBR-treated group and the PR8 group (Figure 7C–I). These results provide direct evidence of the crucial role of MAVS in BBR’s intervention in macrophage pyroptosis mediated by the NLRP3 inflammasome.

## 4. Discussion

The high mutability, pathogenicity, and transmissibility of IAV have positioned it as the primary pathogen driving global influenza pandemics, imposing immense health, economic, and societal burdens [1,41]. In severe cases, influenza often progresses to viral pneumonia, which can severely deteriorate into complications such as acute respiratory distress syndrome, septic shock, and even threaten life [42]. The primary pathogenesis of influenza viral pneumonia stems from excessive inflammatory responses and immunopathological damage triggered by the virus–host interaction post-invasion [7]. This shared mechanism also underlies severe clinical viral pneumonia conditions like COVID-19 and SARS [9,43]. Our previous articles and current research have uncovered in mouse and macrophage models that IAV infection activates the NLRP3 inflammasome, leading to the cleavage of GSDMD and the release of cytokines like IL-1β, subsequently initiating pyroptosis and inflammatory responses (Figure 1 and Figure S1) [36]. This process, intimately linked to viral replication and host cell damage, is a pivotal factor in IAV-induced disease progression. The NLRP3 inflammasome plays a pivotal role in infectious diseases by recognizing both endogenous and exogenous danger signals to promote inflammation; however, its excessive activation can instigate systemic hyperinflammation, further exacerbating damage [44].

Given the crucial roles of innate immune responses and excessive inflammation in viral pneumonia progression, there is broad consensus on investigating virus-induced inflammatory mechanisms and employing anti-inflammatory and immunomodulatory drugs to halt disease progression for clinical prevention and treatment [8]. Inhibitors targeting the NLRP3 inflammasome, such as MCC950, have demonstrated therapeutic efficacy in experimental models, suggesting that targeting the NLRP3 inflammasome and its mediated pyroptosis may emerge as a novel direction for treating viral inflammatory diseases [45,46].

Natural products, endowed with advantages such as low cost, multi-targeting capabilities, and low toxicity, have gradually unveiled their active components and mechanisms of action through advancements in research technology in recent years [47]. This revelation underscores their superiority and vast potential in the treatment of viral infectious diseases [48]. BBR, a natural alkaloid present in various traditional Chinese medicines, has been traditionally employed for the management of gastrointestinal disorders like diarrhea, alongside exhibiting anti-inflammatory, antibacterial, and antiviral properties [28,29,30] and has demonstrated remarkable activity against viral diseases. Contemporary research has validated BBR’s inhibitory effects on influenza virus, respiratory syncytial virus, coronaviruses, and herpes simplex virus [49,50]. This inhibition manifests through reducing cellular damage post-viral infection, lowering viral titers and modulating various stages of the viral replication cycle. Beyond its antiviral prowess, BBR boasts a plethora of pharmacological actions, including anti-tumor, anti-inflammatory, antioxidant, anti-diabetic, and cardio-protective effects, portending extensive application prospects in the treatment of infectious and metabolic diseases [51].

Our study uncovered that BBR significantly suppressed the release of IL-1β and TNF-α in macrophages following IAV infection, with this inhibition exhibiting a dose-dependent pattern. Notably, BBR exhibited optimal anti-inflammatory effects at a high concentration of 16.8 μM, consistent with its previously reported anti-inflammatory characteristics across diverse inflammatory models (Figure 2 and Figure S2) [35,36]. By diminishing the release of these crucial cytokines, BBR may alleviate the excessive inflammatory response triggered by IAV infection, thereby mitigating disease severity. Furthermore, BBR effectively inhibited IAV-induced NLRP3 inflammasome activation, leading to the reduced expression of pyroptosis-related proteins caspase-1 and GSDMD-N. This discovery unravels a novel mechanism of BBR in regulating inflammasome activation. Notably, BBR exhibited comparable efficacy to the specific NLRP3 inhibitor MCC950 in suppressing inflammasome activation and pyroptosis, underscoring its potential in anti-inflammatory therapies (Figure 3).

The activation of the NLRP3 inflammasome involves its translocation within the cell, particularly its crucial localization to mitochondria, which serve not only as energy factories, but also as pivotal regulators of inflammation and cell death [18,45,52]. The findings presented in this study consistently support the high degree of colocalization between NLRP3 and mitochondria, suggesting that mitochondrial dysfunction caused by IAV infection mediates NLRP3-triggered pyroptosis and inflammatory responses (Figure 3D, Figure 5A and Figure S2A). Nevertheless, the therapeutic effects of BBR on the MSU-treated cells were minimal (Figure 4), prompting us to focus on BBR’s regulatory targets on MAVS and mtROS, both of which are crucial molecules in NLRP3 inflammasome activation, facilitating the interaction between NLRP3 and mitochondria. This research revealed that BBR inhibits the transport and binding of NLRP3 to mitochondria by downregulating MAVS expression, thereby blocking inflammasome activation (Figure 6). The intricate relationship between influenza viruses and MAVS stems from MAVS’s pivotal role as a signaling molecule in the host’s innate immune response. Upon the invasion of host cells by RNA viruses such as influenza viruses, their RNA is recognized by RIG-I-like receptors (RLRs) within the cell. These receptors interact with MAVS, triggering the activation of downstream signaling pathways, including TANK-binding kinase 1 (TBK1) and the transcription factor IRF3, ultimately leading to the production of IFN-I. The IFN-I family plays a vital role in inhibiting viral replication and spread [53,54].

However, there are also studies indicating that mtROS generated by the continuous replication of virus can serve as a key to activate MAVS without the involvement of the RLR pathway [55]. In macrophages infected with IAV, viral replication and host cellular stress responses lead to an increase in mtROS production, which subsequently activates the NLRP3 inflammasome. Additionally, excessive ROS may inflict oxidative stress on cells, promoting the activation of multiple inflammatory signaling pathways, ultimately resulting in cellular damage [56]. Notably, BBR is also capable of significantly reducing the mtROS levels induced by IAV infection, an effect comparable to that of mitochondrial antioxidants such as Mito-TEMPO and NAC (Figure 5) [35]. By decreasing mtROS release, BBR inhibits MAVS expression and NLRP3 inflammasome activation, thereby mitigating macrophage pyroptosis and cytokine release. In essence, BBR modulates the MAVS-NLRP3 inflammasome pathway by eliminating mtROS to reduce cellular pyroptosis. In the context of MAVS silencing, since NLRP3 inflammasome activation cannot be triggered through MAVS activation, ROS may activate NLRP3 inflammasome via alternative pathways. Consequently, the levels of LDH, NLRP3 protein, caspase-1-mediated pyroptosis rate, and GSDMD-N expression remain elevated in the PR8 group. In contrast, in BBR-treated macrophages with MAVS silencing, no significant differences were observed in pyroptosis-related molecules compared with the PR8 group (Figure 7). We propose that BBR inhibits pyroptosis by eliminating mtROS, which in turn suppresses the activation of MAVS, NLRP3 inflammasome, and pyroptosis. However, in the absence of MAVS, the effect of BBR may be attenuated.

**Figure 8:**
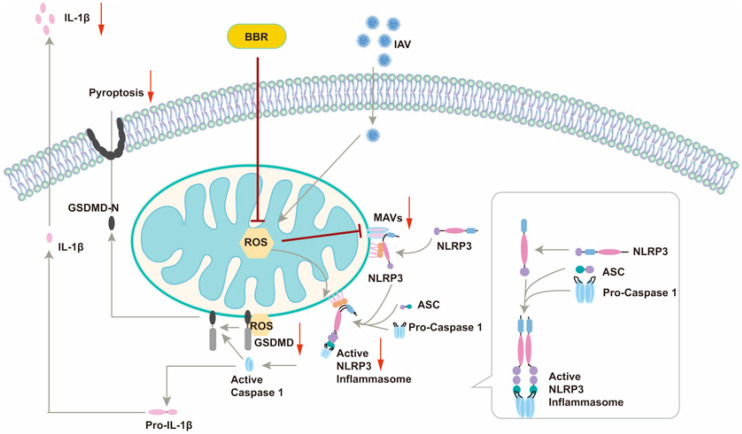
Schematic illustration of the mechanism of BBR intervention on IAV-induced activation of the mtROS-MAVS-NLRP3 inflammasome pathway and GSDMD-mediated pyroptosis in macrophage.

This article unveiled a novel mechanism by which BBR inhibits IAV-induced macrophage pyroptosis, offering a fresh perspective on the intricate interplay between viral infection and the host immune response (Figure 8). By modulating two pivotal molecules, MAVS and mtROS, our study elucidated the multifaceted roles of BBR in suppressing inflammasome activation and pyroptosis, thereby laying a theoretical foundation for the development of multi-target anti-inflammatory drugs. The safety and low toxicity profile of BBR render it an appealing therapeutic candidate, presenting new hope for its clinical application in treating IAV-related infectious diseases, particularly in mitigating inflammatory responses and improving patient outcomes. However, we are cognizant of the remaining key unresolved issues. Firstly, whether BBR’s inhibitory effects on IAV-induced inflammatory responses and cell pyroptosis translate similarly in in vivo models necessitates further validation through animal experiments. Secondly, a deeper understanding of BBR’s precise mechanisms of action, particularly at the cellular and molecular levels, is imperative. Additionally, the dose–response relationship, long-term safety, and potential side effects of BBR require a thorough evaluation in future studies. Addressing these questions will pave the way for the optimal utilization of BBR in clinical settings.

In recent years, the pivotal role of the NLRP3 inflammasome in various inflammatory diseases has garnered significant attention, making the development of its inhibitors a research hotspot [57]. For instance, MCC950 has been proven to be effective in inhibiting NLRP3 activation and reducing the release of inflammatory cytokines in multiple disease models [58]. OLT1177 (dapansutrile), a β-sulfonyl nitrile compound, has demonstrated good safety profiles and has been used to improve inflammation-associated heart failure [59]. Entrectinib, which targets NIMA-related kinase 7 (NEK7), has also been shown to effectively inhibit the activation of the NLRP3 inflammasome [60]. However, the long-term safety of these small-molecule inhibitors in clinical applications remains to be fully established. In addition to small-molecule inhibitors, natural products such as resveratrol and curcumin have been found to have the potential to inhibit the NLRP3 inflammasome [61,62]. Nevertheless, their clinical application prospects are limited by their low bioavailability, short half-life, and poor stability. In contrast, BBR, a natural plant extract, is widely available, cost-effective, and has fewer side effects. In this study, we found that berberine can inhibit NLRP3 inflammasome activation by regulating MAVS. Moreover, berberine has been confirmed to inhibit NLRP3 inflammasome activation through multiple targets including modulating the mTOR/mtROS axis and targeting NEK7 [63,64]. These advantages highlight the significant research and therapeutic potential of berberine in the treatment of NLRP3 inflammasome-related diseases.

## 5. Conclusions

In conclusion, we investigated the impact of BBR on the inflammatory responses and pyroptosis induced by IAV in alveolar macrophages and monocyte-derived macrophages. Through a series of experiments, we discovered that BBR effectively suppresses GSDMD-mediated pyroptosis and inflammatory reactions in IAV-stimulated macrophages. This effect is linked to BBR’s regulation of MAVS and its ability to diminish the release of mtROS. Our study provides a scientific basis for the application of BBR in treating inflammatory diseases caused by IAV infections and also points to directions for future research. With further investigation, we anticipate gaining a deeper understanding of BBR’s mechanisms of action and exploring its potential clinical applications.

## Data Availability

All data generated or analyzed in the current study are included in this published article and its Supplementary Materials. Please contact the corresponding author for details.

## Supplementary Materials

The following supporting information can be downloaded at: https://www.mdpi.com/article/10.3390/v17040539/s1, Table S1: Primers for RT-qPCR used in this study; Table S2: siRNA sequences used in gene knockdown experiments; Figure S1: The mRNA levels of NLRP3, caspase-1, and GSDMD in J774A.1 cells uninfected and infected with the PR8 virus for 6 h, 12 h, or 24 h detected by RT-qPCR; Figure S2: The levels of GSDMD-N in J774A.1 cells detected by immunofluorescence; Figure S3: The mRNA levels of NLRP3, caspase-1, and GSDMD in J774A.1 cells uninfected, infected with PR8, and treated with BBR or MSU detected by RT-qPCR.

## References

[1] Hutchinson E.C. (2018). Influenza Virus. Trends Microbiol..

[2] Javanian M., Barary M. (2021). A brief review of influenza virus infection. J. Med. Virol..

[3] Shinya K., Ebina M. (2006). Avian flu: Influenza virus receptors in the human airway. Nature.

[4] Konig R., Stertz S. (2010). Human host factors required for influenza virus replication. Nature.

[5] Bauer L., Rijsbergen L.C. (2023). The pro-inflammatory response to influenza A virus infection is fueled by endothelial cells. Life Sci. Alliance.

[6] Chen X., Liu S. (2018). Host Immune Response to Influenza A Virus Infection. Front. Immunol..

[7] Gu Y., Hsu A.C. (2019). Role of the Innate Cytokine Storm Induced by the Influenza A Virus. Viral Immunol..

[8] Baek Y.B., Kwon H.J. (2022). Therapeutic strategy targeting host lipolysis limits infection by SARS-CoV-2 and influenza A virus. Signal Transduct. Target. Ther..

[9] Hartshorn K.L. (2020). Innate Immunity and Influenza A Virus Pathogenesis: Lessons for COVID-19. Front. Cell Infect. Microbiol..

[10] Yin H., Jiang N. (2021). Development and Effects of Influenza Antiviral Drugs. Molecules.

[11] Zhang Y., Gao H. (2016). Efficacy of oseltamivir-peramivir combination therapy compared to oseltamivir monotherapy for Influenza A (H7N9) infection: A retrospective study. BMC Infect. Dis..

[12] Wang J., Nikrad M.P. (2012). Innate immune response of human alveolar macrophages during influenza A infection. PLoS ONE.

[13] Vangeti S., Yu M. (2018). Respiratory Mononuclear Phagocytes in Human influenza A virus infection: Their Role in immune Protection and As Targets of the virus. Front. Immunol..

[14] Chen S., Saeed A. (2023). Macrophages in immunoregulation and therapeutics. Signal Transduct. Target. Ther..

[15] Li X., Mara A.B. (2024). Coordinated chemokine expression defines macrophage subsets across tissues. Nat. Immunol..

[16] Lemke G. (2019). How macrophages deal with death. Nat. Rev. Immunol..

[17] Luo L., Wang F. (2024). STAT3 promotes NLRP3 inflammasome activation by mediating NLRP3 mitochondrial translocation. Exp. Mol. Med..

[18] Paik S., Kim J.K. (2021). An update on the regulatory mechanisms of NLRP3 inflammasome activation. Cell Mol. Immunol..

[19] Boal-Carvalho I., Mazel-Sanchez B. (2020). Influenza A viruses limit NLRP3-NEK7-complex formation and pyroptosis in human macrophages. EMBO Rep..

[20] Lei X., Chen Y. (2023). MLKL-Driven Inflammasome Activation and Caspase-8 Mediate Inflammatory Cell Death in Influenza A Virus Infection. mBio.

[21] Coll R.C., Schroder K. (2022). NLRP3 and pyroptosis blockers for treating inflammatory diseases. Trends Pharmacol. Sci..

[22] Zhu B., Wu Y. (2021). Uncoupling of macrophage inflammation from self-renewal modulates host recovery from respiratory viral infection. Immunity.

[23] Yang M., Ma L. (2023). The Extract of Scutellaria baicalensis Attenuates the Pattern Recognition Receptor Pathway Activation Induced by Influenza A Virus in Macrophages. Viruses.

[24] Song D., Hao J. (2020). Biological properties and clinical applications of berberine. Front. Med..

[25] Wang K., Feng X. (2017). The metabolism of berberine and its contribution to the pharmacological effects. Drug Metab. Rev..

[26] Zielinska S., Czerwinska M.E. (2020). Modulatory Effect of Chelidonium majus Extract and Its Alkaloids on LPS-Stimulated Cytokine Secretion in Human Neutrophils. Molecules.

[27] Liang Y., Fan C. (2019). Berberine ameliorates lipopolysaccharide-induced acute lung injury via the PERK-mediated Nrf2/HO-1 signaling axis. Phytother. Res..

[28] Zhang B., Chen M. (2021). Berberine reduces circulating inflammatory mediators in patients with severe COVID-19. Br. J. Surg..

[29] Chen J., Huang Y. (2022). Berberine Ameliorates Inflammation in Acute Lung Injury via NF-kappaB/Nlrp3 Signaling Pathway. Front. Nutr..

[30] Chen L., Liu X. (2023). Berberine Alleviates Acute Lung Injury in Septic Mice by Modulating Treg/Th17 Homeostasis and Downregulating NF-kappaB Signaling. Drug Des. Devel Ther..

[31] Yan Y., Fu Y. (2018). Anti-influenza activity of berberine improves prognosis by reducing viral replication in mice. Phytother. Res..

[32] Varghese F.S., Woudenbergh E. (2021). Berberine and Obatoclax Inhibit SARS-Cov-2 Replication in Primary Human Nasal Epithelial Cells In Vitro. Viruses.

[33] Wu Y., Li J. (2011). In vivo and in vitro antiviral effects of berberine on influenza virus. Chin. J. Integr. Med..

[34] Wang Z., Li K. (2021). A small molecule compound berberine as an orally active therapeutic candidate against COVID-19 and SARS: A computational and mechanistic study. FASEB J..

[35] Liu H., You L. (2020). Berberine suppresses influenza virus-triggered NLRP3 inflammasome activation in macrophages by inducing mitophagy and decreasing mitochondrial ROS. J. Leukoc. Biol..

[36] An C., Wu Y. (2022). Berberine ameliorates pulmonary inflammation in mice with influenza viral pneumonia by inhibiting NLRP3 inflammasome activation and gasdermin D-mediated pyroptosis. Drug Dev. Res..

[37] Gokhale N.S., Sam R.K. (2024). Cellular RNA interacts with MAVS to promote antiviral signaling. Science.

[38] Elliott E.I., Miller A.N. (2018). Cutting Edge: Mitochondrial assembly of the NLRP3 inflammasome complex is initiated at priming. J. Immunol..

[39] Iyer S.S., He Q. (2013). Mitochondrial cardiolipin is required for Nlrp3 inflammasome activation. Immunity.

[40] Subramanian N., Natarajan K. (2013). The adaptor MAVS promotes NLRP3 mitochondrial localization and inflammasome activation. Cell.

[41] Paules C., Subbarao K. (2017). Influenza. Lancet.

[42] Kalil A.C., Thomas P.G. (2019). Influenza virus-related critical illness: Pathophysiology and epidemiology. Crit. Care.

[43] Fung T., Liu D. (2019). Human Coronavirus: Host-Pathogen Interaction. Annu. Rev. Microbiol..

[44] Li Y., Qiang R. (2024). NLRP3 Inflammasomes: Dual Function in Infectious Diseases. J. Immunol..

[45] Swanson K.V., Deng M. (2019). The NLRP3 inflammasome: Molecular activation and regulation to therapeutics. Nat. Rev. Immunol..

[46] Lou S., Wu M. (2024). Targeting NLRP3 Inflammasome: Structure, Function, and Inhibitors. Curr. Med. Chem..

[47] Atanasov A.G., Zotchev S.B. (2021). Natural products in drug discovery: Advances and opportunities. Nat. Rev. Drug Discov..

[48] Ali S.I., Sheikh W.M. (2021). Medicinal plants: Treasure for antiviral drug discovery. Phytother. Res..

[49] Warowicka A., Nawrot R. (2020). Antiviral activity of berberine. Arch. Virol..

[50] Cui Y., Zhang L. (2022). Berberine Inhibits Herpes Simplex Virus 1 Replication in HEK293T Cells. Comput. Math. Methods Med..

[51] Shakeri F., Kiani S. (2024). Anti-inflammatory, antioxidant, and immunomodulatory effects of Berberis vulgaris and its constituent berberine, experimental and clinical, a review. Phytother. Res..

[52] Kelley N., Jeltema D. (2019). The NLRP3 Inflammasome: An Overview of Mechanisms of Activation and Regulation. Int. J. Mol. Sci..

[53] He Q., Huang Y. (2023). MAVS integrates glucose metabolism and RIG-I-like receptor signaling. Nat. Commun..

[54] Chen Y., Shi Y. (2021). MAVS: A Two-Sided CARD Mediating Antiviral Innate Immune Signaling and Regulating Immune Homeostasis. Front. Microbiol..

[55] Dong W., Lv H. (2018). MAVS induces a host cell defense to inhibit CSFV infection. Arch. Virol..

[56] Malireddi R.K., Sharma B.R. (2023). ZBP1 Drives IAV-Induced NLRP3 Inflammasome Activation and Lytic Cell Death, PANoptosis, Independent of the Necroptosis Executioner MLKL. Viruses.

[57] Ma Q. (2023). Pharmacological Inhibition of the NLRP3 Inflammasome: Structure, Molecular Activation, and Inhibitor-NLRP3 Interaction. Pharmacol. Rev..

[58] Wu X., Yang J. (2024). Therapeutic potential of MCC950, a specific inhibitor of NLRP3 inflammasome in systemic lupus erythematosus. Biomed. Pharmacother..

[59] Wohlford G.F., Van T.B. (2020). Phase 1B, Randomized, Double-Blinded, Dose Escalation, Single-Center, Repeat Dose Safety and Pharmacodynamics Study of the Oral NLRP3 Inhibitor Dapansutrile in Subjects with NYHA II-III Systolic Heart Failure. J. Cardiovasc. Pharmacol..

[60] Jin X., Liu D. (2023). Entrectinib inhibits NLRP3 inflammasome and inflammatory diseases by directly targeting NEK7. Cell Rep. Med..

[61] Feng H., Mou S.Q. (2020). Resveratrol Inhibits Ischemia-Induced Myocardial Senescence Signals and NLRP3 Inflammasome Activation. Oxid. Med. Cell Longev..

[62] Benameur T., Frota G.S. (2023). The Effects of Curcumin on Inflammasome: Latest Update. Molecules.

[63] Zhong C., Xie Y. (2024). Berberine inhibits NLRP3 inflammasome activation by regulating mTOR/mtROS axis to alleviate diabetic cardiomyopathy. Eur. J. Pharmacol..

[64] Zeng Q., Deng H. (2021). Berberine Directly Targets the NEK7 Protein to Block the NEK7-NLRP3 Interaction and Exert Anti-inflammatory Activity. J. Med. Chem..

