# Supplementary material for: Berberine Suppresses Influenza A Virus-Triggered Pyroptosis in Macrophages via Intervening in the mtROS-MAVS-NLRP3 Inflammasome Pathway
Source: Viruses. 2025 Apr 7;17(4):539. doi: 10.3390/v17040539 (PMC12030943; doi:10.3390/v17040539)
Supplement: Supplementary file 1 [file viruses-17-00539-s001.zip › viruses-3516530-supplementary.pdf]

Viruses

Supplementary Data

Berberine Suppresses Influenza A Virus-Triggered Pyroptosis in Macrophages via Intervening in the mtROS-MAVS-NLRP3 Inflammasome Pathway

Mengfan Zhao, Di Deng, Hui Liu, Rui Guo, Jun Wu, Yu Hao * and Mingrui Yang *

School of Life Sciences, Beijing University of Chinese Medicine, Beijing 102488, China;
bunianfuhua@163.com (M.Z.); dundee522@163.com (D.D.);
13671081286@163.com (H.L.);
ruiguo9@163.com (R.G.); wujunccg1973@163.com (J.W.)
* Correspondence: yuhao64@sina.com (Y.H.); mingruiyang@bucm.edu.cn (M.Y.)

Supplementary Table S1. Primers for RT-qPCR used in this study.

Genes	Sequences (5' to 3')	Orientation
NLRP3	CCTGACCCAAACCCACCAGT	Forward
NLRP3	TTCTTTCGGATGAGGCTGCTTA	Reverse
Caspase-1	ATGAATCACCAACACCAG	Forward
Caspase-1	CTTGACGCATCCTAATCC	Reverse
GSDMD	AGGGCCACCAAAGCCGGAAGA	Forward
GSDMD	CGATGGAACAAAGCGCAGCAA	Reverse
GAPDH	GGTGAAGGTCGGTGTGAACG	Forward
GAPDH	CTCGCTCCTGGAAGATGGTG	Reverse

Supplementary Table S2. siRNA sequences used in gene knock-down experiments

Names	Sequences (5' to 3')	Orientation
siControl	UUC UCC GAA CGU GUC ACG UTT	Forward
siControl	ACG UGA CAC GUU CGG AGA ATT	Reverse
siMAVS	GCCACCUGUUUCAGUACUATT	Forward
siMAVS	UAGUACUGAAACAGGUGGCTT	Reverse

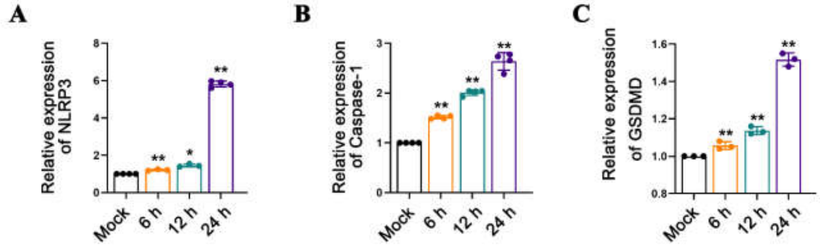

Supplementary Figure S1. The mRNA levels of NLRP3 **(A),** Caspase-1 **(B)** and GSDMD **(C)** in J774A.1 cells uninfected and infected with PR8 virus for 6h, 12h or 24h detected by RT-qPCR.

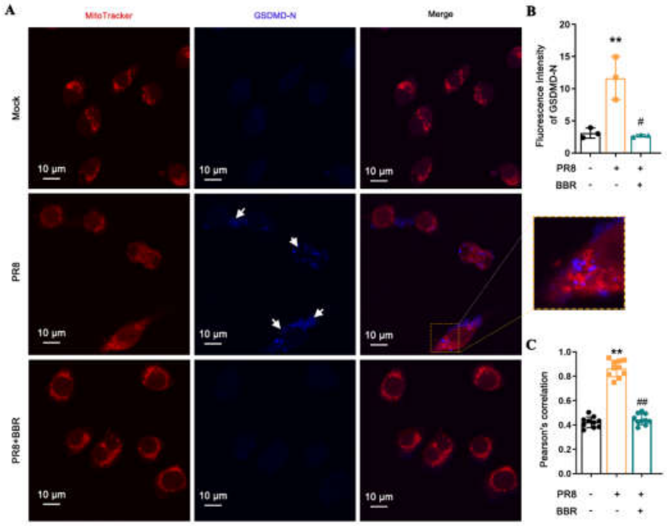

Supplementary Figure S2. The levels of GSDMD-N in J774A.1 cells detected by immunofluorescence. **(A)** Representative images of immunofluorescence staining for mitochondria and GSDMD-N were observed by confocal laser scanning microscope. The mitochondria are shown in red using Mito Tracker Red CMXROS staining; while the GSDMD-N are shown in blue and detected by antibody. The white arrows indicate the fluorescent hub formed by GSDMD-N aggregation. GSDMD-N and mitochondrial colocalization are shown in the dashed box. **(B)** Bar diagram of the quantitative summary for GSDMD-N immunofluorescence. Around sixty cells in each group from three graphs were counted. **(C)** The Pearson correlation coefficient was calculated to assess the colocalization of GSDMD-N with MitoTracker. ** *P*<0.01 *vs* Mock group; $^{\#}$ *P*<0.05 *vs* PR8 group.

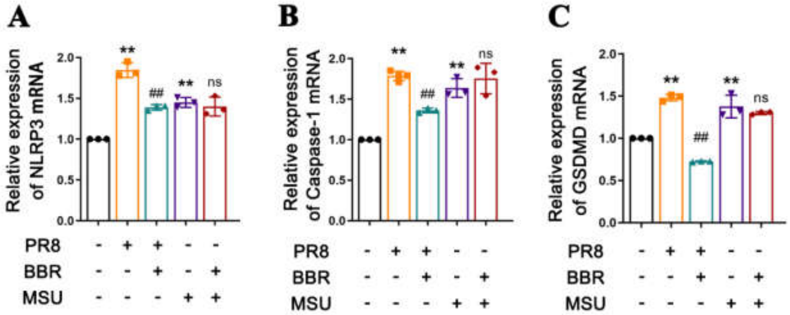

Supplementary Figure S3. The mRNA levels of NLRP3 **(A),** Caspase-1 **(B)** and GSDMD **(C)** in J774A.1 cells uninfected, infected with PR8, and treated with BBR (16.8 μM) or MSU (150 μg/mL) detected by RT-qPCR.
